# Normal Wound Healing and Tumor Angiogenesis as a Game of Competitive Inhibition

**DOI:** 10.1371/journal.pone.0166655

**Published:** 2016-12-09

**Authors:** Irina Kareva, Abdo Abou-Slaybi, Oliver Dodd, Olga Dashevsky, Giannoula Lakka Klement

**Affiliations:** 1 Floating Hospital for Children at Tufts Medical Center, Boston, Massachusetts, United States of America; 2 Mathematical, Computational and Modeling Sciences Center, Arizona State Univ, Tempe, Arizona, United States of America; 3 Massachusetts Institute of Technology, Boston, Massachusetts, United States of America; 4 Dept. of Medical Oncology, Dana−Farber Cancer Institute, Dept. of Medicine, Harvard Medical School, Boston, Massachusetts, United States of America; 5 Pediatric Hematology Oncology, Floating Hospital for Children at Tufts Medical Center, Boston, Massachusetts, United States of America; 6 Sackler School of Graduate Biomedical Sciences at Tufts University, Boston, Massachusetts, United States of America; Yale University School of Medicine, UNITED STATES

## Abstract

Both normal wound healing and tumor angiogenesis are mitigated by the sequential, carefully orchestrated release of growth stimulators and inhibitors. These regulators are released from platelet clots formed at the sites of activated endothelium in a temporally and spatially controlled manner, and the order of their release depends on their affinity to glycosaminoglycans (GAG) such as heparan sulfate (HS) within the extracellular matrix, and platelet open canallicular system. The formation of vessel sprouts, triggered by angiogenesis regulating factors with lowest affinities for heparan sulfate (e.g. VEGF), is followed by vessel-stabilizing PDGF-B or bFGF with medium affinity for HS, and by inhibitors such as PF-4 and TSP-1 with the highest affinities for HS. The invasive wound-like edge of growing tumors has an overabundance of angiogenesis stimulators, and we propose that their abundance out-competes angiogenesis inhibitors, effectively preventing inhibition of angiogenesis and vessel maturation. We evaluate this hypothesis using an experimentally motivated agent-based model, and propose a general theoretical framework for understanding mechanistic similarities and differences between the processes of normal wound healing and pathological angiogenesis from the point of view of competitive inhibition.

## Introduction

A state of chronic inflammation and active angiogenesis, characteristic of many tumors, has led to tumors often being considered “wounds that never heal” [1]. The inflammation accompanying angiogenesis and the associated blood vessel formation, do not necessarily follow tissue planes, but are effective in supplying oxygen, nutrients and in promoting further growth. To understand and potentially modulate tumor angiogenesis, we need to find ways in which tumor angiogenesis differs from normal wound healing. More specifically, we need to identify the mechanisms that lead to normal pruning, stabilization and inhibition of vascular sprouting in wound healing, and why these may be absent in tumor angiogenesis. Both the physiological and pathological variants of angiogenesis are regulated by carefully orchestrated, temporally and spatially controlled signals from surrounding tissues, and it is the sum of these signals the leads to sequential release of stimulators and inhibitors of angiogenesis. We have shown previously that angiogenesis regulators are actively, and against a concentration gradient sequestered in platelets [2], and that stimulators and inhibitors of angiogenesis are differentially released [3, 4].

Platelets are formed by megakaryocytes in the bone marrow, and released into the circulation via pseudopodial extensions [5]. Due to their very short half-life, platelets are continuously renewed and their angiogenesis cargo updated depending on demand. Because for every μL of blood, there are 150,000–450,000 platelets, a typical adult with an average of 5 liters of cardiac output would have 0.750–2.250 trillion circulating platelets at any given time. This fast turnover of large number of circulating platelets containing high concentrations of angiogenesis regulators facilitates a very responsive system for regulating localized angiogenesis at sites of activated endothelium, where platelets form a growth factor rich clots as part of the extracellular matrix (ECM). Platelets, in addition to regulating amounts of angiogenesis regulators at the level of megakaryocyte synthesis [4], use their heavily GAG coated membrane of the open canallicular system, to pick up additional angiogenesis regulators from the tumor microenvironment [2, 6, 7]. During platelet activation, specific surface receptors induce a shape change, extension of lamellipodia and filopodia, and subsequent attachment. The most common platelet aggregation agonists used in clinical laboratories are epinephrine, adenosine di-phosphate (ADP), and thrombin, but *in vivo*, the process is more frequently regulated by fibrin(ogen), (pro)thrombin, von Willebrand factor (VWF) and cleaved products of collagen. Under physiological conditions, platelets are recruited to the wound, and the clot formed at the site effectively traps the enclosed angiogenesis regulators within the ECM matrix, liberating these ECM bound or “trapped” angiogenesis regulators through the action of enzymes such as thrombin [8], heparinase [9], or other locally secreted proteases such as plasminogen activator or collagen.

One of the best described mechanisms of differential release of growth factor from platelets is the proteinase-activated system, presented in 2005 by Li Ma and David Wallace [10]. They showed that the release of growth factors from platelets during the healing of gastrointestinal ulcers was mediated by stimulation of specific proteinase-activated receptors (PARs). The release of pro-angiogenesis proteins, such as vascular endothelial growth factor (VEGF) was triggered by the high affinity thrombin receptor (PAR-1) preferentially activated in low thrombin state, whereas release of angiogenesis inhibitors such as endostatin was triggered by PAR-4, the low affinity receptor triggered in thrombin-rich environment. The gradual transition from low to high concentrations of thrombin is an example of the intricate, highly orchestrated, sequential release of cytokines as the concentrations of proteases in the wound microenvironment change.

The second, less well documented system for orchestrating the sequential release of angiogenesis regulators from platelet-rich ECM at the site of vascular injury is the highly complex system of tissue specific anchors such as heparan sulfate. Throughout this manuscript the term heparin and heparan sulfate are used with the understanding that heparin refers to all heparin moieties in general, i.e. circulating as well as tissue bound, whereas heparan sulfate refers only to tissue bound heparin moiety. Angiogenesis regulators, at least those described to date, have heparin-binding domains and the isoforms often vary in their heparin-binding domain only. This may be a developmentally important evolutionary adaptation determining the tissue specific isoforms. For example, VEGF_121_, a weakly acidic, soluble protein fails to bind heparin and circulates as a decoy, VEGF_165_, the predominant species, is also diffusible, but remains bound to ECM in lung and kidney tissues, VEGF_189_ and VEGF_206_, the more basic proteins binding heparin with greater affinity are sequestered in the extracellular matrix, and VEGF_206_ binds very tightly and has only been identified in human and murine fetal liver cDNA libraries. We suggest that the degree to which angiogenesis regulators bind to GAGs is important in determining the sequence of their release and action.

As we have previously documented using open-ended, mass spectrometry-facilitated comparison of proteomes of platelet from healthy non-tumor-bearing mice and their tumor-bearing counterparts, the presence of a tumor leads to active sequestration angiogenesis regulators in platelets. Upon sequencing the proteins differentially expressed in platelets of tumor bearing mice [2, 6] and humans [11] we found that the most common denominator facilitating the sequestration of proteins in platelets was the ability to bind heparin, suggesting that this was the mechanism of sequestration. Thus, in the setting of a wound the sequence would be as follows: i) VEGF stimulates the initiation of an early vascular sprout (tip cell) [12], ii) the sprout growth and lumenization (stalk cells) are facilitated by bFGF [13, 14]. iii) the proliferation and stabilization of endothelial cells and tube formation is facilitated by medium affinity heparan sulfate binding proteins such as bFGF and platelet derived growth factor (PDGF-B), and finally iv) inhibition of angiogenesis is facilitated by angiogenesis inhibitors such as platelet factor-4 (PF-4) and thrombospondin (TSP-1), which have the highest affinity for HS. The inhibitors of angiogenesis are released last during wound healing and continue to be released under normal physiological conditions. Most of the regeneration and re-establishment of normal tissue architecture during healing occurs by vessel pruning and is accomplished by endogenous inhibitors such as endostatin, tumstatin, thrombospondin-1 and angiostatin, all of which are cleaved products of the collagen matrix [15].

The above-described well-orchestrated system of release of angiogenesis regulators from the platelet-rich ECM is based on cell-cell interactions. Specific signals produced during wound healing by cell-cell interactions initiate, promote or terminate formation of new blood vessels. However, in order to do so, angiogenesis regulators need to be bound to tissue glycosaminoglycans (GAGs) such as heparan sulfate (HS) on the cells’ surface [16–18]. This structural anchoring to the tissue facilitates receptor signaling [19] and enables cell-cell feedback triggering or inhibiting secretion of enzymes such as heparinase from fibroblasts and other stromal elements. In contrast, increased heparinase secretion degrades HS, effectively destroying the tissue anchors and increasing the turnover of angiogenesis regulators. For as long as tissue injury exists, the up-regulation of GAG formation and angiogenesis continue, but with healing, as increasing amounts of thrombin and scar tissue are deposited, HS is down-regulated and the system turns off. It should be stressed here that the ‘normal physiological state’ of post-natal angiogenesis is ‘off’ except for placental implantation and wound healing. The absence of postnatal angiogenesis is further supported by the finding that the concentration of angiogenesis inhibitors such as TSP-1 and PF-4 in platelets is orders of magnitudes higher than that of stimulators [11], and by the predominance of angiogenesis inhibitors in tissues because most of the HS sites are firmly occupied by high-affinity angiogenesis inhibitors such as TSP-1 and PF-4.

This quiescence is altered in a cancer setting. The oncogene activation of tumor stroma leads to inflammation, activation of proliferative pathways and the constantly advancing tumor edge does not permit accumulation of thrombin, down-regulation of HS, and binding of angiogenesis inhibitors. Because stronger bonds are needed for high affinity binding, it requires more time and more stable environment than that provided by an advancing edge. This manuscript presents biological experiments evaluating the manner in which heparin affinity of angiogenesis regulators affects the order of growth factor elution from platelets, and offers a conceptual framework, which uses an agent-based model to simulate the data *in silico*. The proposed theoretical framework suggests that wound healing is governed by a competitive interaction of high and low affinity angiogenesis regulators for tissue binding sites.

## Materials and Methods

### *In vitro* experiments

We have previously documented the presence of angiogenesis regulators in normal healthy platelets [11], and in platelets of tumor bearing animals [2, 6]. To demonstrate the sequential release of angiogenesis stimulators and inhibitors from a thrombus, we used NaCl elution of proteins from a column of activated human platelets (a clot). Platelets were collected from healthy human volunteers using direct venipuncture into standard sodium citrate (1/9 v/v) vacutainer tubes. Individuals with history of cancer, inflammatory disease, diabetes or non- steroidal anti-inflammatory drug use were excluded from the study. The protocol for platelet collection (IRB# 11096) was specifically approved by the Tufts Medical Center Institutional Ethics Review Board), and signed written consent was obtained for each of the healthy volunteers used for this study.

We used salt elution as a surrogate for tissue proteases because the relationship between increasing concentration of salt and growth factor affinity is linear, and independent of other environmental conditions such as temperature or acidity. This is a standard elusion method used in the majority of studies investigating heparin-binding growth factors [20–22]. A platelet clot was made from 1mL of platelet rich plasma, and incubated with heparin affinity beads in a heparin sepharose column (GE healthcare). The platelet clot and heparin column was allowed to equilibrate for 3 hours at 37°C. The column was then washed using with 10 mL of physiological saline [0.154M NaCl] (i.e 10 volumes of the original volume of beads in the column, which is 1 mL). The proteins were eluted with 1 mL NaCl of increasing molar concentrations between 0.154 and 4M. In this experiment, the NaCl acts by disrupting the ionic interactions between heparan sulfate and the corresponding growth factor. Under physiological conditions, heparinase would cleave the heparan sulfate chain to which the growth factor is bound.

**Fig 1:**
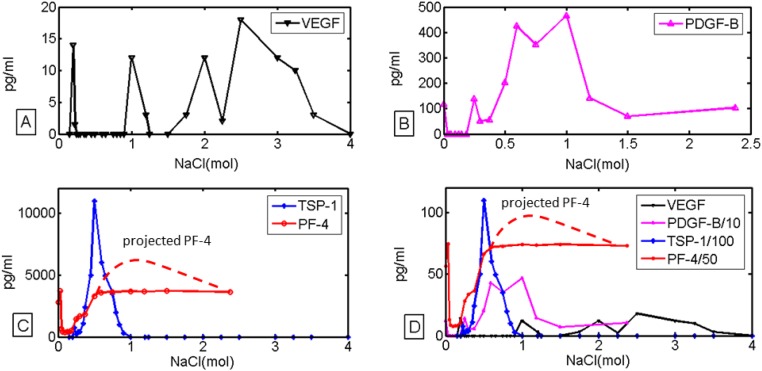
Release of angiogenesis regulators from platelets is sequential. The activity of tissue proteases such as heparinase, which is an enzyme that cleaves heparan sulfate from the cell membranes, is simulated by increased concentrations of NaCl. As shown in a, b and c, higher concentrations of NaCl are required for the release of growth factors with higher heparan sulfate affinities, going from VEGF (a) to PDGF-B (b) to PF-4 and TSP-1 (c). The observed plateau in PF-4 was due to the detection limit of the PF-4 ELISA. The predicted shape of the curve in the absence of ELISA detection limit is shown as a dotted red line. Fig 1D shows an overlay of each protein with PDGF-B divided by 10, PF-4 divided by 50 and TSP-1 divided by 100. Each experiment was repeated 3 times in technical duplicate.

Each 1mL elution was then quantified using an ELISA assay (R and D Systems Inc) for each corresponding protein, VEGF, PF-4, TSP-1 and PDGF-BB. The observed plateau in PF-4 was due to the detection limit of the PF-4 ELISA. The experiments were repeated three times with technical duplicates for each faction. Negative values due to noise or as a consequence of approximations done using linear regression were corrected to 0. Platelets contain angiogenesis inhibitors in quantities that are many fold higher than those of angiogenesis stimulators. In order to accommodate these widely discrepant amounts on a single graph, we had to divide the values for PF-4 by 50, the values of TSP-1 by 100, and the values of PDGF-B by 10. The results are reported in Fig 1.

To confirm the relevance of the experiment to physiological conditions we repeated the experiment using a limited dose response curve of heparinase (see S1 Fig). Even though the action of heparinase is much more dependent on temperature, acidity, oxygen availability, as well as on other tissue-dependent factors, the results were qualitatively consistent with results obtained using NaCl.

### Agent-based model

The overreaching goal of the endeavor was to understand the role of platelets associated angiogenesis regulators in normal physiological angiogenesis and its pathological counterpart. We explored the processes from the premise that because platelet angiogenesis regulators remain associated with the clot, they get incorporated in the ECM, and their effect is regulated by competition for HS binding sites. In this framework, should the stimulators out-compete the inhibitors, one would expect to see unrestrained angiogenesis.

To evaluate the hypothesis, we constructed an agent-based in Netlogo 5.0.2, a freely available agent-based modeling platform, where a 2-dimensional 51x51 lattice represents space. Each grid on the lattice represents a discrete microenvironment, the properties of which are given below. The model allows for 2601 microenvironments. Cells occupy coordinates in continuous space; more than one cell can occupy a single microenvironment. Time is modeled in discrete steps. We focus on three broad classes of growth factors in this model, and the classes are characterized by their affinities for HS. More specifically, we refer to stimulators of angiogenesis (VEGF) as low-affinity growth factors (LA), to vessel stabilizers (PDGF-B) as medium affinity growth factors (MA), and to inhibitors (PF-4, TSP-1) as high-affinity growth factors (HA).

The specific assumptions used for this model for each agent and for the microenvironments are as follows:

#### Properties of cells

cells of the stroma are populated on the grid randomly. Cells are non-motile and are characterized by a parameter that determines the number of binding sites to which the growth factors can attach. The number of binding sites is taken from a random distribution, with a predefined mean and variance. The number of the free binding sites is decreased as they become occupied by a growth factor, and increased, when the growth factor is cleaved by heparinase.

#### Properties of low affinity (LA) growth factors

Low affinity (LA, VEGF) cytokines are the first type of growth factors to be released into the microenvironment at the beginning of the simulation. LA are motile and move randomly throughout the grid. LA are characterized by 2 parameters: probability of attachment to a free binding site, and sensitivity to heparinase; LA become cleaved and release the binding sites at the lowest concentration of heparinase compared to other growth factors. Once they encounter a cell, they evaluate the availability of free binding sites. If a binding site is available, they attach with a certain pre-defined probability, and become cleaved depending on the concentration of heparinase in the corresponding microenvironment. When they attach, the decrease the number of binding sites available to other growth factors. Number of free binding sites is restored when Las are cleaved.

#### Properties of medium affinity (MA) growth factors

Medium affinity (MA, PDGF) cytokines are the second type of growth factors to be released into the microenvironment at the beginning of the simulation. MAs are motile and move randomly throughout the grid. MAs are also characterized by 2 parameters: probability of attachment to a free binding site, and sensitivity to heparinase; MAs become cleaved and release the binding sites at the higher concentration of heparinase compared to LAs but lower than HAs. Once MAs encounter a cell, and free binding sites are available, they attach with a certain pre-defined probability, and become cleaved depending on the concentration of heparinase in the corresponding microenvironment. When they attach, the number of binding sites available to other growth factors is reduced. The number of free binding sites is restored after MAs are cleaved.

#### Properties of high affinity (HA) growth factors

High affinity (MA, PF-4 and TSP-1) cytokines are the third and last type of growth factors to be released into the microenvironment at the beginning of the simulation. Like LA and MA, HA are motile and move randomly throughout the grid. HA are also characterized by 2 parameters: probability of attachment to a free binding site, and sensitivity to heparinase; HA become cleaved and release the binding sites at highest concentration of heparinase compared to other growth factors: once they become attached, they take a long time to become cleaved. Once HA encounter a cell, they evaluate the availability of free binding sites. If a binding site is available, they attach with a certain pre-defined probability, and become cleaved depending on the concentration of heparinase in the corresponding microenvironment. When they attach, the decrease the number of binding sites available to other growth factors. Number of free binding sites is restored after HA become cleaved.

#### Properties of the microenvironments

**Fig 2:**
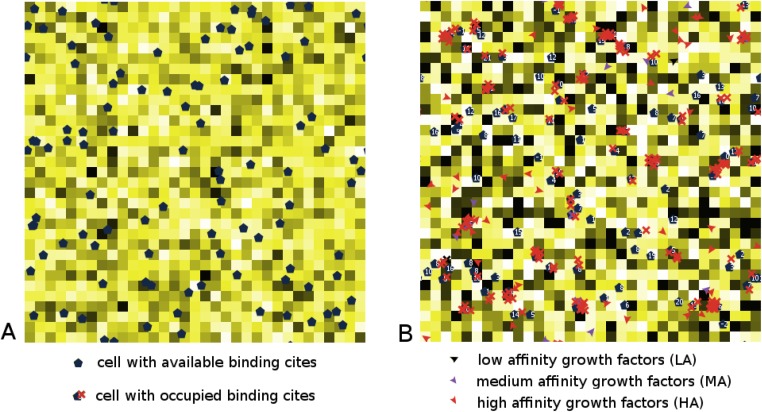
A snapshot of the proposed agent-based model. (A) Model initialization. The grid is populated by cells, which are characterized by pre-determined number of binding sites. The color changes in the microenvironments represent heparinase concentration. Darker patches correspond to higher concentrations of heparinase; lighter patches correspond to lower levels of heparinase. (B) After the simulation has begun, the growth factors LA, MA and HA mode randomly throughout the grid. Once they encounter a cell with free binding sites (red crosses), they occupy them, and thereby decreasing the number of available sites (numbers by the cell agents decreases, reflecting the number of available sites). Growth factors become cleaved depending on the current concentration of heparinase in the corresponding microenvironment. The NetLogo code for this model is attached in Supporting Information.

Each microenvironment is characterized by some amount of heparinase, different for each patch. Heparinase is removed and replenished in the microenvironments continuously over time to simulate heparinase flux in the tissue microenvironment. The concentration of heparinase is visually depicted in the simulation in yellow; higher levels of heparinase in the microenvironment are represented by darker shades of yellow (see Fig 2). The NetLogo program code is attached in Supporting Information (see S1 Code). A homeostatic balance of heparinase is maintained over time throughout the grid.

The model is initialized after the growth factors have been sequentially released from platelets (not included in this model), starting from LA, followed by MA, and finally by HA. At each time step, the following sequence of events is realized:

1. Cytokines are released into the tumor microenvironment (once in the beginning for simulation of normal wound healing; at each time step in case of simulation of a tumor)
2. Cytokines move randomly throughout the grid. Once they encounter a cell, they evaluate availability of binding sites.
3. If a binding site is available, the growth factor attaches, thereby reducing the number of available binding sites.
4. Depending in the type of cytokine and on the amount of heparinase in the corresponding microenvironment, the cytokine remains bound or becomes cleaved. LA become cleaved at lowest concentration of heparinase, followed by MA, and finally followed by HA, which become cleaved only at highest levels of heparinase.
5. Heparinase is randomly removed or replenished from each microenvironment.
6. In the presence of a tumor, LA, MA and/or HA may become replenished as well, depending on each simulated case. Addition of LA and MA creates competition for binding sites for HA.

In this model we do not incorporate formation of blood vessels as a result of such signaling as it is outside the scope of this particular investigation. This model is proof-of-concept, aimed to evaluate the hypothesis that presence of competition for binding sites may cause continuous presence of growth factors that are capable of constant pro-angiogenesis signaling.

### Parameter estimation

In the experiment described above, we demonstrated that different concentrations of NaCl, which served as a proxy for heparinase, cleave distinct angiogenesis regulatory proteins from the column. Angiogenesis stimulators (VEGF) have the lowest affinity for NaCl and thus heparinase, followed by blood vessel stabilizers (PDGF), which have medium affinity for NaCl, followed by angiogenesis inhibitors (PF-4 and TSP-1), which have the highest affinity for heparinase. Since NaCl is a proxy for heparinase, we are not able to retrieve exact parameter values for affinities of angiogenesis regulators for heparinase; however, we can use the extrapolated relationships and relative values of parameters for the purposes of our proof-of-concept model. Specifically, the rate of detachment of angiogenesis stimulators (VEGF) from binding sites will be lower compared to rates of detachment of blood vessel stabilizers (PDGF), which in turn will be lower than that of angiogenesis inhibitors (PF-4 and TSP-1).

## Results

We have shown in earlier studies that the majority of platelet associated angiogenesis regulating proteins are retained in the clot upon platelet aggregation and activation [2]. This earlier work [2] challenged the widely accepted assumption that platelets degranulate and thus release proteins *en ma*sse. We now hypothesize that this is a direct consequence of the varying heparin sulfate-binding affinities of stimulators and inhibitors of angiogenesis, and that there is a specific order to the release of angiogenesis regulators from the platelet clots. Using increasing concentration of NaCl as a surrogate for increasing concentration of tissue proteases, our results indicate that platelet-associated angiogenesis regulators may be eluted in sequence corresponding to their heparin-binding affinities. A similar sequential release has been previously reported for tissues but not in the context of platelets [20]. More specifically, VEGF, a low-affinity angiogenesis stimulator that induces sprout formation, is released first (Fig 1a), followed by medium-affinity growth factors, such as PDGF-B (Fig 1b), and subsequently by highest-affinity inhibitors, such as PF-4 (Fig 1c). That is, as the concentrations of stimulators peak, the concentration of inhibitors predominates and angiogenesis returns to the physiologically dominant state, i.e. quiescence of angiogenesis (Fig 1d). The need for tissue- VEGF concentration gradients may be the main reason that higher organisms have tissue-dependent expression of specific VEGF isoforms, each of which differs not in the main protein core, but in their ability to bind heparin. We can appreciate two peaks of VEGF in Fig 1 (one at 0.154 M NaCl, and another one at 1M NaCl), most likely due to VEGF_165_ and VEGF_189_ isoforms. Unfortunately, because present ELISA assays cannot distinguish between VEGF_165_, VEGF_189_ and VEGF_206_ an experimental confirmation is not possible. In contrast, the two peaks of PF-4 in Fig 1c are more likely caused by the abundance of PF-4 in platelets, and a wash out of its excess at low NaCl concentrations, as no isoforms of PF-4 have been described.

### Simulation of normal wound healing

**Fig 3:**
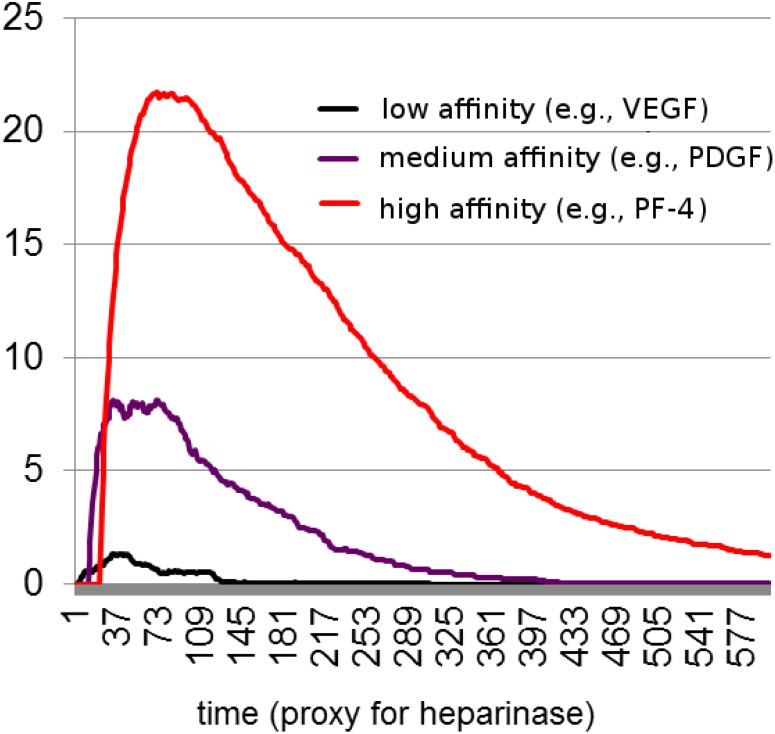
Simulation of cytokine release during normal wound healing. Angiogenesis regulators bound to a heparin sulfate moiety in platelet clots are eluted using incremental concentrations of sodium chloride as a surrogate for tissue proteases. The experimentally observed release of growth factors appears to suggest that angiogenesis regulators are released in order of their affinity for heparan sulfate, with VEGF having the lowest affinity, and PF-4 and TSP-1 the highest.

Our first goal was to replicate using an agent-based modeling method, the experimentally observed pattern of sequential release of cytokines, observed in Fig 1. The starting values for low-affinity (LA), medium-affinity (MA) and high-affinity (HA) used for the simulation were taken from [11]. Because experimentally reported physiological concentrations of angiogenesis inhibitors (HA) are several orders of magnitude higher, we have adjusted these values for the simulation. This computational adjustment does not, from model construction point of view, qualitatively affect the predicted dynamics. The only predicted change in the outcome is the time of the simulation, because more HA growth factors would bind and release over time. We ran 10 simulations for the initial parameter set, and data was collected by counting the number of each type of bound cytokines at each time point. The number of each cytokine was averaged and the summary given in Fig 3. All of the data for individual curves for all ten simulations can be found in supplementary materials (see S2–S8 Figs for individual simulations). As one can see in Fig 3, the model reproduces a pattern consistent with expected behavior of angiogenesis regulators during normal wound healing. Importantly, this pattern was reproduced using a set of few basic assumptions about growth factors with different affinities competing for available binding sites.

### Cytokine competition during tumor angiogenesis

A number of studies report increased secretion of lower and medium affinity growth factors (e.g. VEGF and PDGF-B) in the tumor microenvironment [20]. It is also well documented that expression of glycosaminoglycans such as heparan sulfate is increased at the sites active injury [23] or tumor [16]. Within a competition-based framework proposed here, we hypothesize that increased inflow and turnover of lower-affinity growth factors leads to higher-affinity growth factors (inhibitors) being outcompeted for binding sites leading to inability of inhibitors to bind and restrain angiogenesis. The HS-tightly binding angiogenesis inhibitors such as PF4 and TSP1 may also not be released from the platelet clot as they require more time and protease activity to be released. Thus, while the process of new tumor vessel formation would commence in the same way as vessel formation in wound healing process, it would not terminate by release of inhibitors. The model is supported by the observation that the levels of PF-4, a tumor angiogenesis inhibitor are decreased with tumor progression [6].

**Fig 4:**
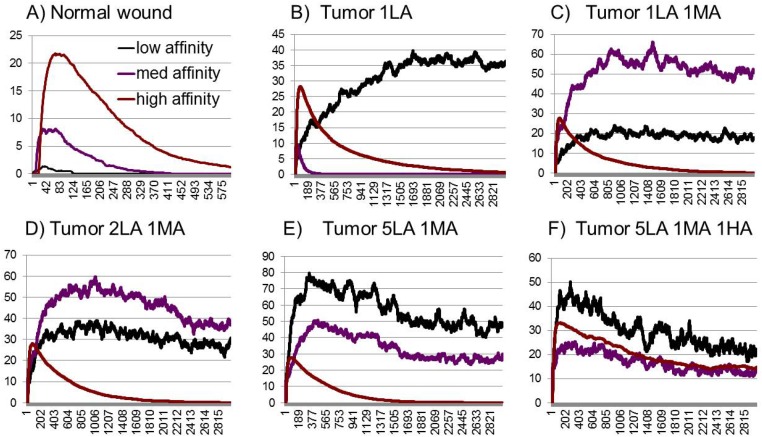
Summary of simulations for normal and pathological angiogenesis. The simulation of cytokine profiles in normal wound healing (A) is distinctly different from the cytokine profiles during tumor angiogenesis (B-F). In order to simulate tumor angiogenesis, a number of scenarios were explored: The release of 1 additional low affinity cytokine released into the model microenvironment per run (B); 1 additional low affinity (LA) and 1 medium affinity (MA) (C); 2 LA and1MA (D); 5LA and 1MA (E); and 5LA, 1MA and 1HA (F). The combination of continuous presence of LA and MA may be suggestive of continued formation of new, but leaky vessels.

In order to evaluate this hypothesis, we modify the model to allow for a continuous inflow of low and medium affinity growth factors from the tumor cells, or from stromal elements in response to hypoxia. We run five different sets of numerical experiments and compare the predicted curves to the ones that correspond to the process of normal wound healing, given in Fig 3. More specifically, we introduce the following modifications: a) 1 additional LA per run, b) 1 additional LA and 1 MA, c) 2LA 1MA, d) 5LA, 1MA e) 5LA 1MA 1HA. The values were chosen arbitrarily, for the purposes of evaluation of the predicted effects of different relative rates of inflow of growth factors of various types. The results of the simulations can be seen in Fig 4, and details of the simulations are available in supplementary materials (see S3–S8 Figs for individual simulations).

As can be seen in these examples, a continuous release of lower and medium affinity cytokines leads to inhibitors being out-competed from binding sites and unable to restrain angiogenesis. The relative rates of inflow can be varied along with variations of growth factors based on tumor or tissue specificity. A specific sub-study may be modeling tumor dynamics emphasizing differences between actively growing and dormant tumors.

## Discussion

A number of mathematical models have previously been proposed to describe pathological angiogenesis, using both equation-based and agent-based models (see, for instance [24–29]). However, to our knowledge, no model has yet described the process of normal wound healing turning to pathological angiogenesis from the point of view of competition of angiogenesis regulators for binding sites, a gap that we fill in this work.

In 1986, Dvorak suggested that tumors can be viewed as wounds that do not heal [1]. He outlined the many similarities between normal wound healing process and tumor angiogenesis, and suggested that an understanding of normal vessel formation in wound healing would enable interpretation of the abnormalities of blood vessel formation in tumor angiogenesis. Wound healing/angiogenesis is a multistep process involving the uptake of angiogenesis regulators by platelets, trapping of regulator-rich platelet clots in wounded tissues, and a well-orchestrated secretion of positive and negative regulators of angiogenesis from the platelet clots. As documented previously, platelet clots trap angiogenesis regulators [2], and release them in response to tissue proteases [3]. The angiogenesis regulators are anchored in tissues to surface glycosaminoglycans (GAGs) such as heparan sulfate (HS) [18], and their turnover is facilitated by continuous secretion of heparinase and other tissue proteases by stromal elements. This sequence allows for sprouting, lumenization and regulation of blood vessels. The early attachment of lowest affinity angiogenesis regulators such as VEGF (angiogenesis stimulators) initiates sprout formation. Growth factors with medium affinity, such as PDGF-B and bFGF (stabilizers) continue the process by stimulating endothelial cell proliferation, tube formation, and vessel stabilization [13, 14, 30]. Cytokines with highest affinity (PF-4 and TSP-1) are inhibitors that promote collagen cleavage and vessel pruning, effectively terminating the wound healing process [7]. This is supported by the many fold excess of angiogenesis inhibitors in platelets of normal human subjects [11], and explains the default postnatal “off” state of postnatal angiogenesis.

It appears that vessel stabilization and pruning does not occur in tumors [31], resulting in a continuous formation of immature, leaky blood vessels. We propose that this immature vessel phenotype is a result of increased inflow of stimulators and stabilizers such as VEGF and PDGF-B flooding the HS binding sites. Once the HS sites are occupied, inhibitors, such as PF-4 or thrombospondin, which require more time in order to tightly bind, are prevented from binding and terminating angiogenesis. To evaluate the implications of this hypothesis, we constructed an imitative agent-based model, which incorporates three types of growth factors characterized by their ability to bind HS as low (VEGF), medium (PDGF-B) and high (PF-4) affinity growth factors. The growth factors are motile and seek to bind to GAG on the surface of cells they encounter, and vacate these binding sites as the activity of heparinase in the local microenvironment increases. Despite setting only a minimal set of assumptions, we were able to reproduce a pattern of behavior that corresponds to the expected behavior of angiogenesis regulators during normal wound healing (see Fig 3). This theoretical model provides a very plausible mechanistic explanation for the early part of a normal wound healing process, as well as for pathological tumor angiogenesis.

A modification of the model, where an excess of low and medium affinity growth factors is created (such as would be the case in malignant tumors), confirmed that such a cytokine storm would preclude inhibitor binding and a successful inhibition of blood vessel formation. The best way to envisage this type of interaction is as a game of musical chairs between growth factors competing for binding sites. The theoretical model provides a novel framework for understanding the sequential release and binding of angiogenesis regulators in wound healing and tumor angiogenesis from the point of view of *competitive inhibition*. While both processes commence the same way, the continuous inflow of stimulators and stabilizers that accompanies early wound healing is not turned off in tumor invasion, because the advancing edge of the growing tumor, does not allow time for tissue proteases to create pro-inhibitory microenvironment. Even though these angiogenesis regulators have distinctly separate receptors, anchoring to HS is a pre-requisite for effective signaling [19], and the binding at the glycan level enables this competitive inhibition.

### Model implications

We are working from our earlier findings that angiogenesis regulators are stored in platelet alpha-granules, and released from platelet clots in response to tissue proteases. The model, as presented here, does not provide information about the source, character or relative proportions of the specific cytokines. An example of protease-facilitated growth factor release from platelets is the thrombin Proteinase Activated Receptor (PAR) system. In this system lower affinity growth factors are released in response to stimulation of PAR-1 (the high affinity thrombin receptor stimulated even in low thrombin state), and higher affinity growth factors are released upon stimulation of PAR-4 [3, 10].

There is a number of other local controls of growth factor release from platelet clots, such as for example acidosis, oxygen tension and temperature [32–34]. The microenvironment of tumors is known to have sites with low pH due to up-regulated glycolysis [35, 36], and decreased pH leads to dramatic decrease of thrombin or collagen-induced secretion of vascular endothelial growth factor [33]. Because pH is just one of the regulators of physiological functions [37], the overall effect of release of inhibitors vs stimulators of angiogenesis is very heterogeneous even within a tumor microenvironment.

**Fig 5:**
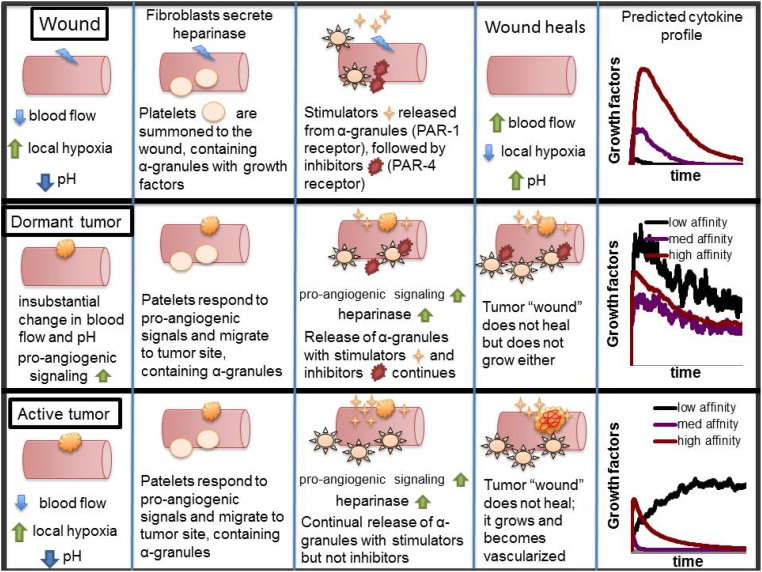
Schematic representation of the proposed mechanism underlying normal wound healing, pathological angiogenesis and differences in both dormant and progressive tumors.

Within the context of normal wound healing, one can entertain the following: During injury, loss of vascular integrity leads to deficient perfusion, increased rescue glycolysis, and lowering of pH, providing a physiological signal for angiogenesis. Platelets, as early responders to tissue injury deliver platelet-associated angiogenesis regulators (both stimulators and inhibitors), and due to a relative absence of thrombin, heparinase or other tissue proteases, angiogenesis stimulators are released first. The blood vessel damage and poor perfusion leads to tissue ischemia and acidosis further de-activating PAR-4, and preventing release of angiogenesis inhibitors. This mechanism would allow for enhanced formation of new blood vessels until pH is normalized, PAR-4 re-activated, inhibitors released, and angiogenesis switched off. The hypoxia and low pH associated with tumor microenvironment effectively simulate this physiological state of normal wound healing. The observation that low pH dramatically reduces thrombin or collagen-induced secretion of vascular endothelial growth factor and endostatin [33] may also provide a plausible explanation for the differences in cytokine profiles between progressive and dormant tumors. Dormant tumors are small enough to avoid central necrosis, and thus regions of increased acidosis, hyperthermia and hypoxia, and they retain a physiological homeostasis with normal balance of predominance of angiogenesis inhibitors. These considerations are summarized in Fig 5.

The agent-based model described in this manuscript provides a contextual framework for understanding the differences between normal wound healing and unrestrained angiogenesis in tumors. The mechanistic explanation provided by a competitive inhibition model may allow experimental and therapeutic exploration that would give a competitive advantage to angiogenesis inhibitors.

## Supporting Information

S1 Fig Heparinase elution of growth factors from a thrombus depends on growth factor affinities. TSP-1 has the highest affinity and cannot be eluted even with 1M heparinase. However, elution curves do confirm the differential elution rates between FGF, PDGF and TSP-1, with FGF requiring the lowest amount of heparinase to be eluted, followed by PDGF and TSP-1.
(PNG)

S2 Fig Predicted elution dynamics of low (LA), medium (MA) and high (HA) affinity angiogenesis regulators from platelet clots in normal wound healing. (TIFF)

S3 Fig Predicted elution dynamics for low (LA), medium (MA) and high (HA) affinity growth factors over time over time in tumor microenvironment. Simulated tumor: 1 additional LA per run.
(TIFF)

S4 Fig Predicted elution dynamics for low (LA), medium (MA) and high (HA) affinity growth factors over time over time in tumor microenvironment. Simulated tumor: 1 additional LA, 1MA per run.
(TIFF)

S5 Fig Predicted elution dynamics for low (LA), medium (MA) and high (HA) affinity growth factors over time over time in tumor microenvironment. Simulated tumor: 2 additional LA, 1MA per run.
(TIFF)

S6 Fig Predicted elution dynamics for low (LA), medium (MA) and high (HA) affinity growth factors over time over time in tumor microenvironment. Simulated tumor: 5 additional LA, 1MA per run.
(TIFF)

S7 Fig Predicted elution dynamics for low (LA), medium (MA) and high (HA) affinity growth factors over time over time in tumor microenvironment. Simulated tumor: 5 additional LA, 1MA and 1HA per run.
(TIFF)

S8 Fig Predicted elution dynamics for low (LA), medium (MA) and high (HA) affinity growth factors over time over time in tumor microenvironment. Simulated tumor: 5 additional LA, 1MA and 2 HA per run.
(TIFF)

S1 Code "NetLogo code of the proposed agent-based model of normal wound healing and pathological angiogenesis as a game of competitive inhibition. (NLOGO)
