# Supplementary figures and images for: Normal Wound Healing and Tumor Angiogenesis as a Game of Competitive Inhibition
Source: PLoS One. 2016 Dec 9;11(12):e0166655. doi: 10.1371/journal.pone.0166655 (PMC5147849; doi:10.1371/journal.pone.0166655)

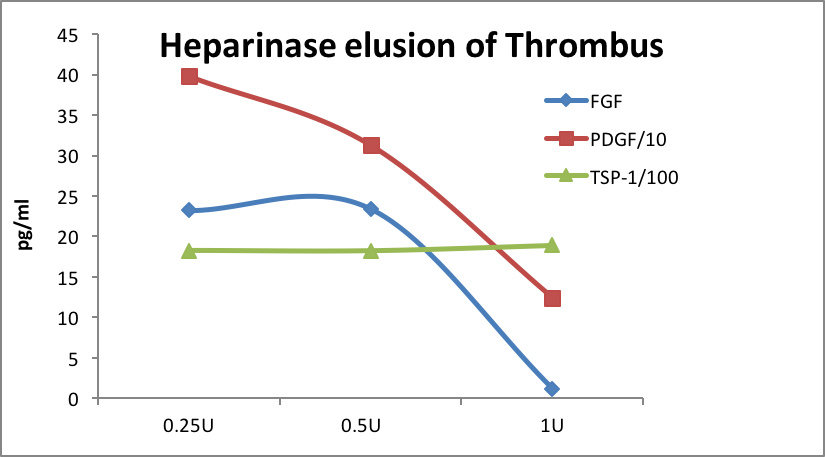

Supplement: S1 Fig — TSP-1 has the highest affinity and cannot be eluted even with 1M heparinase. However, elution curves do confirm the differential elution rates between FGF, PDGF and TSP-1, with FGF requiring the lowest amount of heparinase to be eluted, followed by PDGF and TSP-1. (PNG) [file pone.0166655.s001.png]

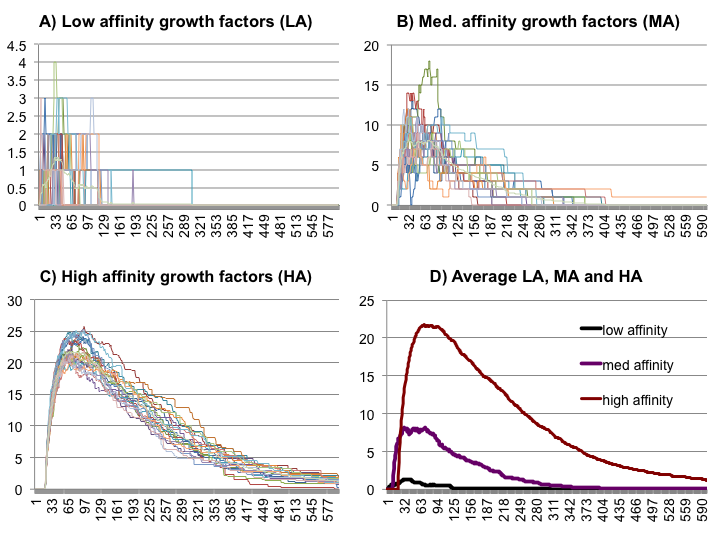

Supplement: S2 Fig — (TIFF) [file pone.0166655.s002.tiff]

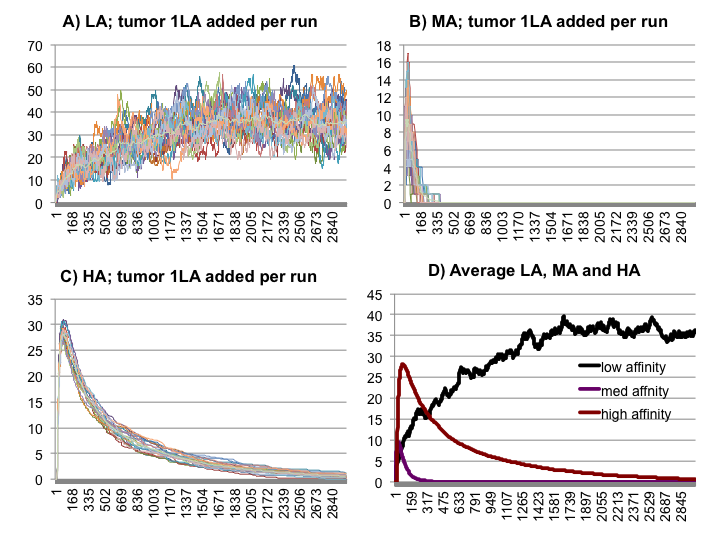

Supplement: S3 Fig — Simulated tumor: 1 additional LA per run. (TIFF) [file pone.0166655.s003.tiff]

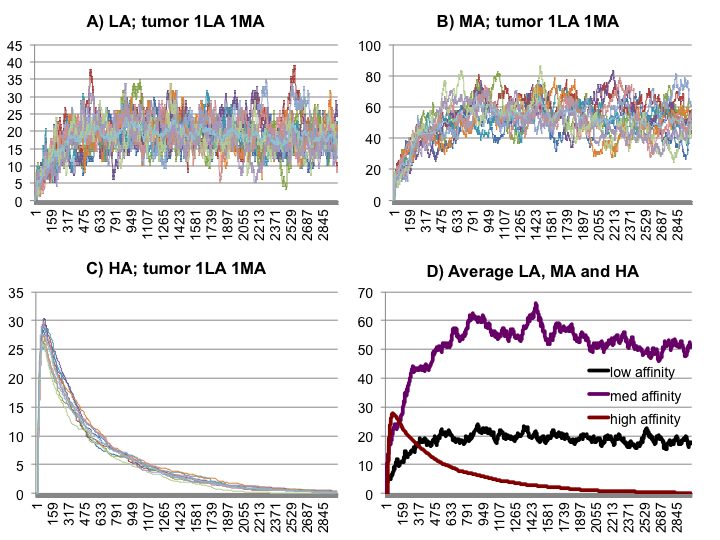

Supplement: S4 Fig — Simulated tumor: 1 additional LA, 1MA per run. (TIFF) [file pone.0166655.s004.tiff]

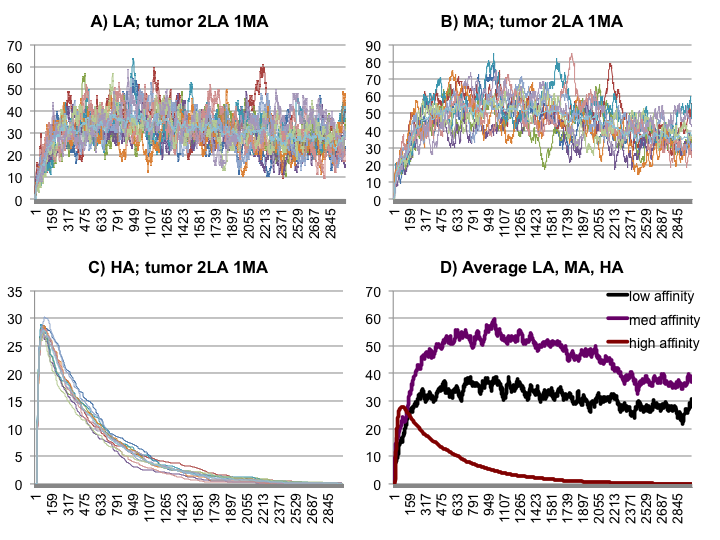

Supplement: S5 Fig — Simulated tumor: 2 additional LA, 1MA per run. (TIFF) [file pone.0166655.s005.tiff]

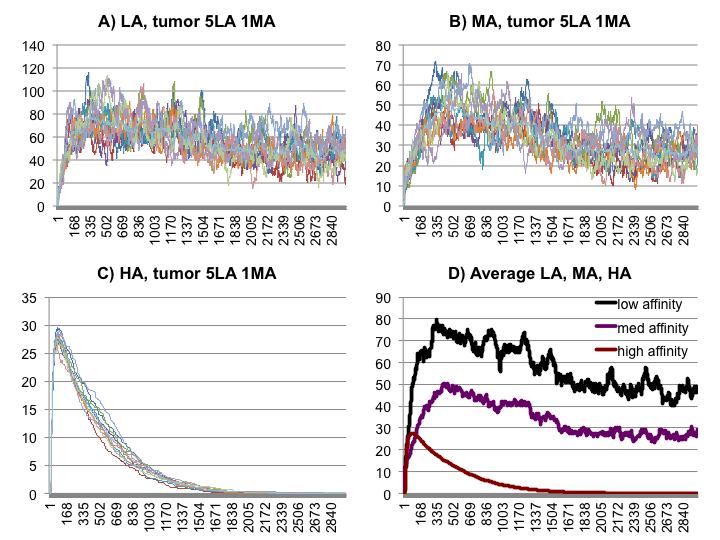

Supplement: S6 Fig — Simulated tumor: 5 additional LA, 1MA per run. (TIFF) [file pone.0166655.s006.tiff]

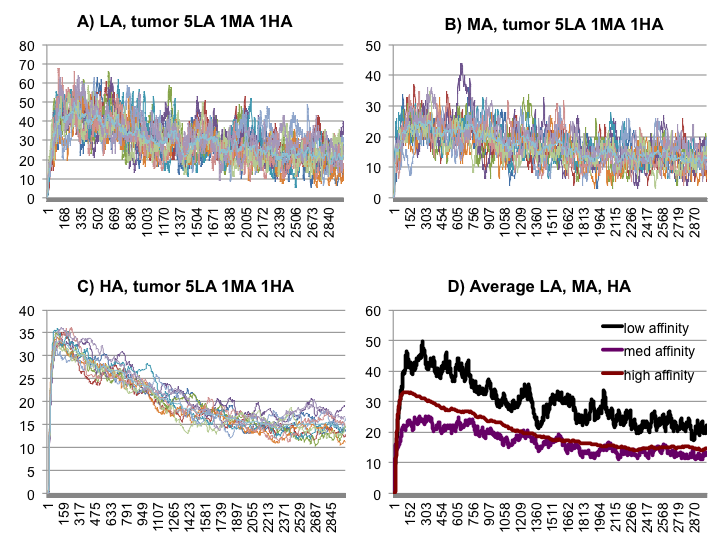

Supplement: S7 Fig — Simulated tumor: 5 additional LA, 1MA and 1HA per run. (TIFF) [file pone.0166655.s007.tiff]

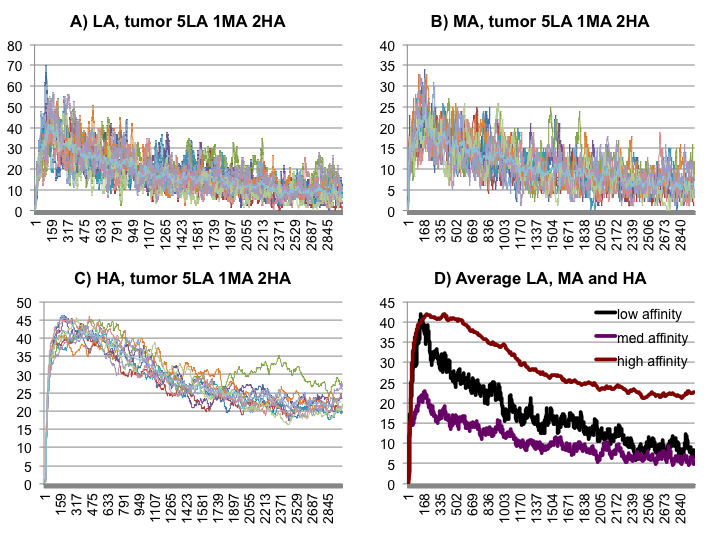

Supplement: S8 Fig — Simulated tumor: 5 additional LA, 1MA and 2 HA per run. (TIFF) [file pone.0166655.s008.tiff]
